# Androgen receptor splice variant 7 detected by immunohistochemical is an independent poor prognostic marker in men receiving adjuvant androgen-deprivation therapy after radical prostatectomy

**DOI:** 10.1186/s40364-021-00276-x

**Published:** 2021-03-31

**Authors:** Wei Ouyang, Yucong Zhang, Gongwei Long, Guoliang Sun, Man Liu, Fan Li, Chunguang Yang, Xing Zeng, Jun Yang, Xiao Yu, Zhihua Wang, Zheng Liu, Wei Guan, Zhiquan Hu, Shaogang Wang, Xiaming Liu, Heng Li, Hua Xu, Zhangqun Ye

**Affiliations:** 1 Department of Urology, Tongji Hospital, Tongji Medical College, Huazhong University of Science and Technology, Wuhan, 430030 China; 2 Hubei Institute of Urology, Tongji Hospital, Tongji Medical College, Huazhong University of Science and Technology, Wuhan, China; 3 Department of Geriatrics, Tongji Hospital, Tongji Medical College, Huazhong University of Science and Technology, Wuhan, China

**Keywords:** Prostate cancer, AR-V7, Adjuvant hormonal therapy, Cohort study, Prognosis

## Abstract

**Background:**

To evaluate the predictive value of AR-V7 expression detected by immunohistochemical (IHC) in the prognosis of prostate cancer patients receiving adjuvant hormonal therapy (AHT) following radical prostatectomy (RP).

**Methods:**

We retrospectively collected data of 110 patients with prostate cancer receiving RP, followed by AHT, from Tongji hospital. IHC analysis of AR-V7 expression was performed in a retrospective cohort.

**Results:**

In total, 110 patients were enrolled, of whom 21 patients (19.1%) were AR-V7-positive and 89 patients (80.9%) were AR-V7-negative. No significant differences in baseline characteristics were found between the two groups. AR-V7-positive patients had shorter progression-free survival (PFS) (HR: 4.26; 95% CI, 1.55 to 11.68; *P* = 0.003), shorter cancer-special survival (CSS) (HR: 22.47; 95% CI, 2.912 to 173.4; *P* = 0.003) and shorter overall survival (OS) (HR: 6.61; 95% CI, 1.40 to 31.20; *P* = 0.017) compared to AR-V7-negative patients. In multivariate analysis, AR-V7 is an independent risk factor for shorter PFS (HR, 3.76; 95% CI, 1.63 to 8.70; *P* = 0.002), shorter CSS (HR: 9.17; 95% CI, 1.48 to 55.56; *P* = 0.017) and shorter OS (HR: 4.81; 95% CI, 1.28 to 17.86; *P* = 0.020).

**Conclusion:**

The presence of AR-V7 in prostate cancer tissue is independently associated with an unfavorable prognosis for PFS, OS and CSS in patients who received AHT.

**Supplementary Information:**

The online version contains supplementary material available at 10.1186/s40364-021-00276-x.

## Introduction

It was estimated that there were almost 1.3 million new cases of prostate cancer and 359,000 associated deaths worldwide in 2018, ranking as the second most frequent cancer and the fifth leading cause of cancer death in men [1]. It is the most frequently diagnosed cancer among men in over one-half (105 of 185) of the countries around the world. In China, prostate cancer is one of the most common and deadly male malignant tumors [2]. Although prostate-specific antigen (PSA) testing has been popular in prostate cancer screening, some patients were firstly diagnosed with locally advanced disease.

Radical prostatectomy (RP) and radiotherapy (RT) are regarded as first-line treatment for localized prostate cancer. However, some patients with high-risk prostate cancer experienced biochemistry recurrence (BCR) rapidly after curative treatment [3–8]. Therefore, RP should be regarded as a part of multi-modal therapy for high-risk localized and locally advanced prostate cancer, and adjuvant treatment, such as hormonal therapy, radiotherapy, or chemotherapy, are usually required after RP for those patients. Some previous published studies have already demonstrated that adjuvant hormonal therapy (AHT) after RP was beneficial to patients with nodal metastases [9, 10]. In a randomized clinical trial (RCT), a total of 309 patients diagnosed with stage pT3–4 pN0 were included [11]. All patients were divided randomly into 2 groups. The study group received adjuvant flutamide 750-mg once daily after RP while the control group only received RP. After a median follow-up of 6.1 years, patients in the study group experienced longer progression-free survival (PFS) compared with patients in the control group. However, the difference between the two groups was insignificant for overall survival (OS). Another clinical analysis showed the efficacy of bicalutamide as an adjuvant treatment after RP for locally advanced, nonmetastatic prostate cancer, and concluded that bicalutamide could prolong the PFS versus standard care alone, but not OS [12].

Androgen Receptor Splice Variant 7 (AR-V7) was an abnormally spliced mRNA isoform of the androgen receptor (AR). It can drive the expression of androgen-responsive genes by androgen independent pathway because of the C-terminal ligand-binding domain deficiency and transcriptional active N-terminal domain existence [13–15]. Some pre-clinical and clinical studies have shown the association between AR-V7 expression and the resistance to androgen receptor signal pathway inhibitor (ARSi) such as enzalutamide or abiraterone in castration-resistant prostate cancer (CRPC). The AR-V7 expression was associated with shorter PFS and OS in CRPC patients [16–20]. Our previous study demonstrated that AR-V7 expression was also associated with worse prognosis in hormone-sensitive prostate cancer (HSPC) patients who received androgen deprivation therapy (ADT) [21]. However, whether the expression of AR-V7 in prostate cancer tissue has a prognostic effect on the treatment outcomes for patients received AHT after RP remains unknown. AHT with luteinizing hormone releasing hormone analogs (LHRHa, goserelin, leuprorelin, triptorelin) or anti-androgen (AA, flutamide, bicalutamide) or combined androgen blockade (CAB) were administered after RP, according to doctor’s decision in routine clinical practice as per the 2014 version of the Chinese Guidelines for Prostate Cancer [22]. Our study aims to assess the expression of AR-V7 as a prognostic factor for the response to AHT in nonmetastatic HSPC (nmHSPC).

## Methods

### Patients and tissues

Our study retrospectively collected 110 prostate cancer patients who underwent RP and extended pelvic lymph node dissection (ePLND) at Tongji Hospital, Tongji Medical College, Huazhong University of Science and Technology, during years 2010–2017. The inclusion criteria for patients in the study were: 1) age ≥ 18 years; 2) histological confirmation of prostate adenocarcinoma; 3) high-risk prostate cancer (Gleason score ≥ 8 or preoperative serum PSA ≥ 20 ng/mL) or locally advanced prostate cancer (pT3/pT4, N0M0 and any T, N1M0) or positive surgical margins (R1); and 4) immediate administration with AHT after surgery. Patients were excluded if they initial received additional concurrent anticancer therapy (RT, chemotherapy). All included patients received AHT. The AHT included medical castration (LHRHa), combined with anti-androgens (bicalutamide etc.). Informed consent was obtained from all participants.

### Study design and assessments

This retrospectively study aimed to evaluate the ability of baseline (before AHT) AR-V7 status (positive vs. negative) to predict the treatment outcomes of AHT after RP. This study was carried out in accordance with the ethical standards of the Helsinki Declaration and approved by the Tongji Hospital of Huazhong University of Science and Technology (Wuhan, China) ethics review committee (reference TJ-IRB20170801), and registered in the Chinese Clinical Trial Registry (NO ChiCTR1800015334, http://www.chictr.org.cn/).

Follow-up assessments were retrospectively collected and included PSA measurements, prostate ultrasound scans, computed tomography (CT) of the chest, abdomen, and pelvis, and technetium-99 m bone scanning. The AR-V7 status and clinical data were evaluated in a blind and independent manner. All immunohistochemical slides were examined and scored by two experienced pathologists, who were blinded to all clinical data. If the Immune-Reactive Score differed between two investigators, a third investigator evaluated the tissue sections, and the average score was recorded.

### Clinical outcomes

The primary outcome was PFS, which was defined as the time from surgery to disease progression. Disease progression including BCR and clinical or radiographic progression. BCR was defined as a PSA increased for two consecutive measurements and PSA level ≥ 0.2 ng/ml for localized disease and was defined as an increase in the PSA level by 25% or more above the nadir (and by ≥2 ng/ml), with confirmation four or more weeks later for lymphatic or distant metastasis, according to PCWG3 criteria [23]. Clinical or radiographic progression was defined as symptomatic progression (worsening disease-related symptoms or new cancer-related complications), radiographic progression (the appearance of new lesions: either two or more new bone lesions on bone scan or a soft tissue lesion [according to the Response Evaluation Criteria in Solid Tumors] [15, 24]).

The secondary end points were OS and cancer-special survival (CSS). OS was defined as the time from surgery to die for any reason. CSS was defined as the time from surgery to die of prostate cancer.

### Immunohistochemistry and evaluation

IHC staining was performed on surgical specimens to assess AR-V7 expression (rabbit monoclonal, ab198394, Abcam, Cambridge, UK, 1:150 dilution), androgen receptor full length (AR-FL) expression (rabbit polyclonal, ab133273, Cambridge, UK, Abcam, 1:100 dilution) by Bond Polymer Refine Detection System (Leica Biosystems Newcastle, Newcastle upon Tyne, UK). We also reviewed all reported AR-V7 IHC studies (see Supplementary Table 1). The AR-V7 positive rates of nmHSPC vary substantially among the different studies [21, 25–29] (range 1.6% from 91.8%), which may because of the differences in the TN stage, ethnicity and antibody. Some studies in fact to the large extent suggest that ab198394 (EPR15656 or EP343) is reliable in prostate cancer tissue IHC [21, 25, 26]. The omission of the primary antibody with phosphate-buffered saline served as a negative control for this detection system. The status of AR-V7 expression was assessed by using an Immune-Reactive Score that included the intensity and quantity of cells stained [29–31]. The staining intensity including negative, weak, moderate or strong, which was scored as 0, 1, 2, 3, respectively. The percent positivity was scored as 0 (< 1%); 1 (1–10%); 2 (11–50%); 3 (51–80%) and 4 (> 81%). The final immune-reactive scores were presented as the product of intensity and quantity (range 0–12). Immune-reactive scores < 2 were considered negative and scores ≥2 were considered positive.

### Statistical analysis

All statistical analyses were performed with SPSS, v.22 (IBM, Armonk, NY) and GraphPad Prism v.7 (La Jolla, CA). Continuous variables were presented as median (range) or mean (standard deviation) and categorical data were presented as number (proportion). Patients’ clinical and pathological characteristics were compared by the Student’s t test for continuous variables and the Chi square test of continuity correction for categorical variables. PFS, OS, and CSS were estimated by the Kaplan-Meier method and compared by log-rank test. Both univariate and multivariate Cox regression analysis models were used to compare hazard ratio (HR) and evaluate the predictive role of all covariates for PFS, OS, and CSS. All statistical tests were two sides, *P* < 0.05 was considered significant. Five-years’ survival rates were compared by Z-test.

## Results

### Patients’ baseline characteristics

**Fig. 1:**
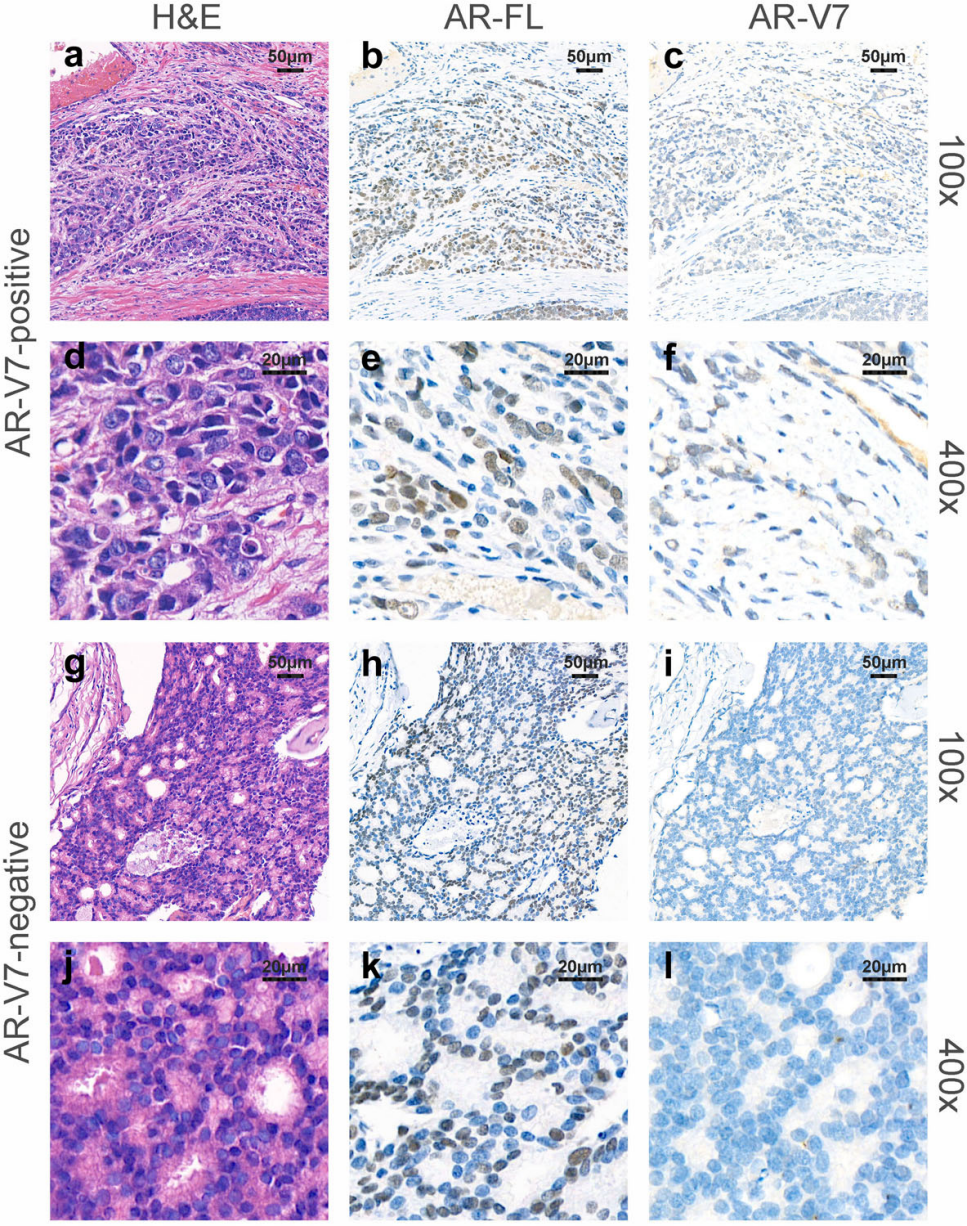
Representative immunohistochemical (IHC) and hematoxylin–eosin (H&E) staining. **a**, **d**, **g**, **j** H&E staining; **b**, **e**, **h**, **k**) immunohistochemical staining for N-terminal androgen-receptor full-length (AR-FL) and (**c**, **f**, **i**, **l**) immunohistochemical staining for AR-V7 in two representative tissue samples. The first and second rows are AR-V7-positive from consecutive tissue sections, and the third and fourth rows are AR-V7-negative. Original magnification: × 100 (**a**, **b**, **c**, **g**, **h**, **i**) and × 400 (**d**, **e**, **f**, **j**, **k**, **l**)

Among these 110 patients, 78 were censored, including 4 received RT, 8 drop out and 66 because of the end of follow-up period. The baseline characteristics of included patients are shown in Table 1. Twent-one patients (19.1%) were AR-V7 positive and 89 (80.9%) were AR-V7 negative. Representative IHC staining is shown in Fig. 1. Baseline characteristics were comparable between these two groups, including age (*P* = 0.598), Gleason scores (*P* = 0.748), PSA (*P* = 0.368), T stage (*P* = 0.555), N stage (*P* = 0.444), prostate volume (*P* = 0.105), prostate-specific antigen density (PSAD) (*P* = 0.368), surgical margin (*P* = 0.811) and follow-up time (*P* = 0.964). The median follow-up time among AR-V7-positive patients and AR-V7-negative patients were 57.6 (range: 41.1 to 76.5) and 57.6 (range: 33.7 to 83.3) months, respectively.

**Table 1:** Baseline clinicopathological characteristics of all enrolled patients

Characteristics	Total	AR-V7 positive	AR-V7 negative	*P*
No. of patients (%)	110	21	89	
Age (mean, SD, year)	66.02 (6.12)	65.38 (5.35)	66.17 (6.31)	0.598
Gleason score (n, %)				
≤ 7	77 (65.25)	14 (66.67)	56 (62.92)	0.748
≥ 8	41 (34.75)	7 (33.33)	33 (37.08)	
Pathological T stage (n, %)				
2	75 (63.56)	14 (66.67)	51 (57.30)	0.555
3	33 (27.97)	5 (23.81)	32 (35.96)	
4	10 (8.47)	2 (9.52)	6 (6.74)	
Pathological N stage (n, %)				
0	83 (70.34)	17 (80.95)	62 (69.66)	0.444
1	35 (29.66)	4 (19.05)	27 (30.34)	
Surgical Margin (n, %)				
R0	99 (83.90)	17 (80.95)	74 (83.15)	0.811
R1	19 (16.10)	4 (19.05)	15 (16.85)	
Preoperative TPSA (mean, SD, ng/ml)	44.46 (58.80)	58.32.44 (81.76)	41.19 (52.02)	0.368
Prostate volume (mean, SD, cm3)	71.74 (34.02)	60.90 (18.15)	74.30 (36.39)	0.105
PSAD (mean, SD, ng/ml/cm3)	0.73 (1.17)	1.29 (2.29)	0.60 (0.63)	0.186
Follow-up time (mean, SD, months)	57.57 (10.56)	57.67 (9.24)	57.55 (10.90)	0.964

*SD* Standard Deviation, *TPSA* Total prostate-specific antigen, *PSAD* Prostate-specific antigen density. *P*-values less than 0.05 are highlighted in bold

### AR-V7-positive is associated with worse prognosis after AHT

**Fig. 2:**
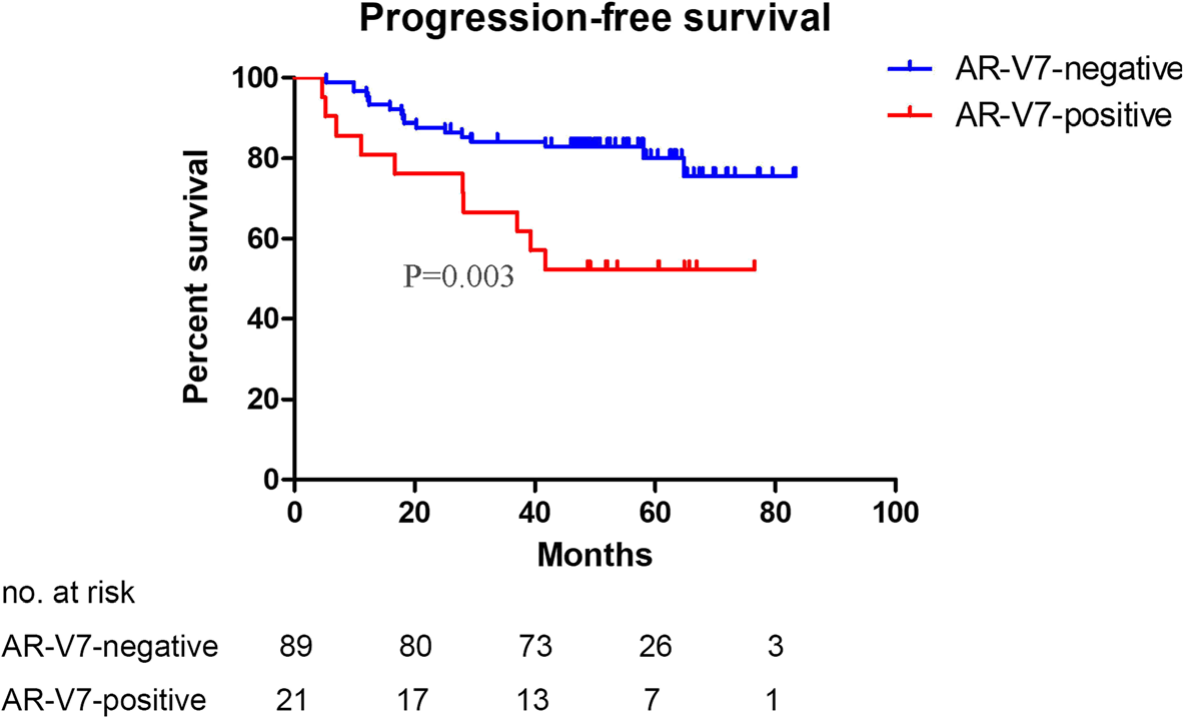
Kaplan–Meier analysis of PFS according to AR-V7 status. PFS: progression-free survival

Kaplan-Meier analysis showed AR-V7-positive patients had shorter PFS (HR: 4.26; 95% CI, 1.55 to 11.68; *P* = 0.003) than AR-V7-negative patients (Fig. 2). The median PFS in AR-V7-negative patients was not reached (range: 5.3–89.6 months), whereas 58.6 months (range: 4.6–76.5 months) in AR-V7-positive patients. The results of the univariate Cox analyses are shown in Supplementary Table 2 and the results of multivariable Cox analyses are shown in Table 2. The multivariable Cox model showed that the expression of AR-V7 is an independent risk factor for shorter PFS (HR, 3.76; 95% CI, 1.63 to 8.70; *P* = 0.002) after adjusting for age, TN stage, Gleason score and total PSA (Table 2). The five-year survival rate of PFS among AR-V7-positive was lower than that of AR-V7-negative patients (52.4% vs 80.1%, *P* = 0.004).

**Table 2:** Multivariate Cox analyses of PFS, OS and CSS in patients received AHT

Variables	Outcomes	Multivariate analysis	
		HR (95% CI)	*P*-value
Age at diagnosis (ref: < 65.0 years) Age ≥ 65.0 years	PFS	1.17 (0.50–2.71)	0.717
	OS	3.04 (0.62–14.93)	0.169
	CSS	4.27 (0.45–40.00)	0.204
Gleason score at diagnosis (ref: 6≤ G ≤ 7) Gleason score ≥ 8	PFS	0.62 (0.25–1.58)	0.317
	OS	1.70 (0.39–7.46)	0.483
	CSS	3.02 (0.43–20.83)	0.265
Total prostate-specific antigen (ng/ml) (ref: < 20) PSA ≥ 20	PFS	1.06 (0.46–2.48)	0.887
	OS	1.32 (0.29–6.06)	0.721
	CSS	1.20 (0.12–11.76)	0.878
Pathological T stage at diagnosis (ref: T_2_) T3,4	PFS	3.53 (1.39–9.01)	**0.008**
	OS	0.72 (0.15–3.50)	0.682
	CSS	0.98 (0.10–9.35)	0.987
Pathological N stage at diagnosis (ref: N0) N1	PFS	1.75 (0.73–4.18)	0.210
	OS	1.92 (0.46–8.06)	0.371
	CSS	0.81 (0.08–8.62)	0.862
AR-V7-positive (ref: AR-V7-negative)	PFS	3.76 (1.63–8.70)	**0.002**
	OS	4.81 (1.28–17.86)	**0.020**
	CSS	9.17 (1.48–55.56)	**0.017**
R1(ref: R0)	PFS	2.02 (0.80–5.08)	0.135
	OS	1.79 (0.38–8.47)	0.465
	CSS	0.51 (0.04–7.35)	0.620

*AHT* adjuvant hormonal therapy, *PSA* prostate specific antigen, *PFS* progression-free survival, *OS* overall survival, *CSS* cancer-special survival. *P*-values less than 0.05 are highlighted in bold

**Fig. 3:**
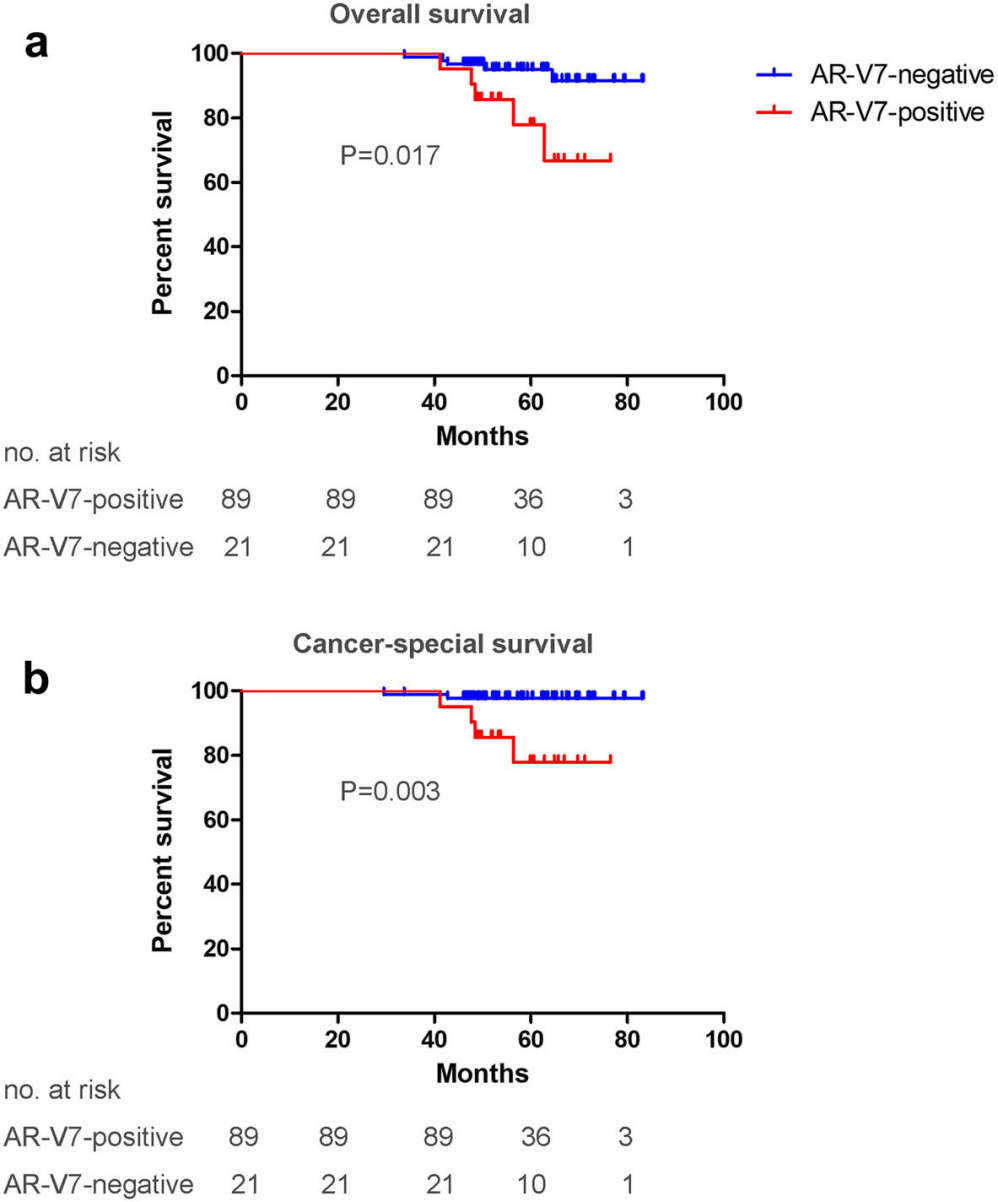
Kaplan–Meier analysis of OS (**a**) and CSS (**b**) according to AR-V7 status. OS: overall survival; CSS: cancer-special survival

Overall, OS (HR: 6.61; 95% CI, 1.40 to 31.20; *P* = 0.017) and CSS (HR: 17.07; 95% CI, 2.35 to 124.07; *P* = 0.005) were significantly shorter among AR-V7-positive patients than AR-V7-negative patients (Fig. 3). During the follow-up period, 10 people died, of whom 5 patients were AR-V7-positive. The multivariable Cox model showed that the expression of AR-V7 is the only independent risk factor for shorter OS (HR: 4.81; 95% CI, 1.28 to 17.86; *P* = 0.020) and shorter CSS (HR: 9.17; 95% CI, 1.48 to 55.56; *P* = 0.017) after adjusting for age, TN stage, Gleason score and total PSA (Table 2). The five-year survival rates of OS and CSS among AR-V7-positive and AR-V7-negative patients were 77.9% vs 94.9% (*P* = 0.009) and 77.9% vs 97.7% (*P* = 0.012), respectively.

**Fig. 4:**
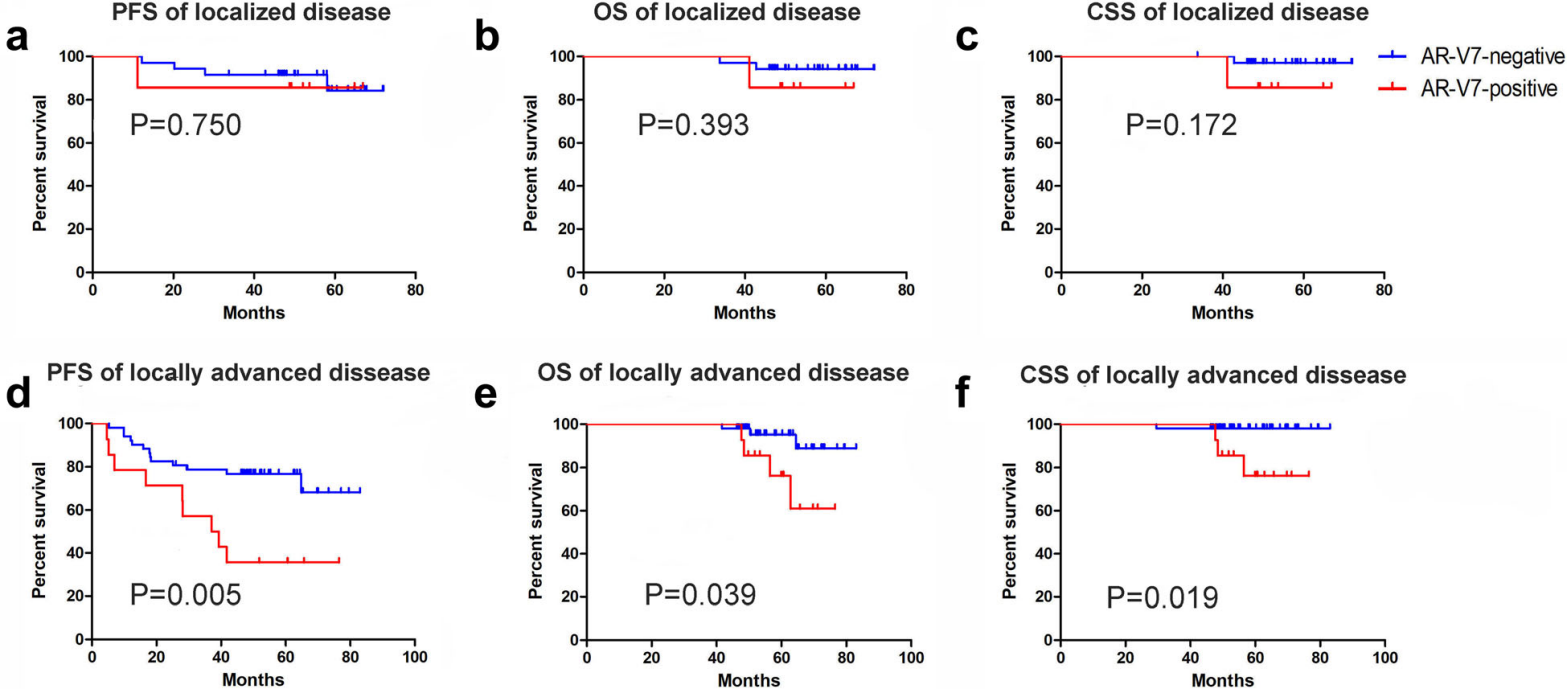
Kaplan–Meier analysis of PFS, OS and CSS according to AR-V7 status in patients stratified by localized (**a**, **b**, **c**) or locally advanced disease (**d**, **e**, **f**). PFS: progression-free survival; OS: overall survival; CSS: cancer-special survival

We further stratified patients into localized and locally advanced disease. In localized disease, Kaplan–Meier analyses indicated that the differences of PFS (*P* = 0.705), OS (*P* = 0.393) and CSS (*P* = 0.172) among patients with different AR-V7 status were not significant (Fig. 4a, b, c). In locally advanced disease, Kaplan–Meier analyses indicated that PFS was lower in AR-V7-positive patients than in AR-V7-negative patients (median PFS: 38.18 months vs. undefined, *P* = 0.005) (Fig. 4d, e, f). The median OS (*P* = 0.039) and CSS (*P* = 0.019) in AR-V7-positive patients were also shorter than that in AR-V7-negative patients.

## Discussion

Since the dependence of prostate cancer on androgen signaling firstly discovered by Huggins and Hodges [32], hormonal therapy has been considered as the standard treatment for locally advanced and metastatic prostate cancer. AHT aimed to improve the long-term survival of patients with high-risk localized prostate cancer, positive surgical margin, and pathologically positive lymph nodes [24, 33]. Although some retrospective studies showed that AHT cannot provide significant prognostic benefits in patients with minimal nodal [12, 34], a RCT demonstrated that early AHT provides significantly CCS and OS improvement in high-risk prostate cancer [10]. The finding suggested RP plus postoperative AHT was an important component of multimodal strategies for high-risk prostate cancer. Unfortunately, though AHT could control the development of the disease for several years, most of these patients would experience recurrence and even death [35], which is consistent with our survival surveillance data.

Quite a few scholars believe that one reason for the resistant to ADT is the generation of AR splice variants. Until now, more than 30 distinct AR splice variants have been identified [36]. Among these variants, AR-V7 is the most common one [13, 14, 37]. AR-V7 is a truncated androgen-receptor protein, which retains the trans-activating N-terminal domain while lacks the C-terminal ligand-binding domain [13, 15]. It can promote the activation of target genes irrespective of serum androgen levels, leading to the development and growth of prostate cancer [29]. Therefore, the expression of AR-V7 may indicate a poor response and prognosis for ARSi or ADT [38]. Our previous retrospective study indicated that AR-V7 expression in newly diagnosed prostate cancer is intimately correlated with the prognosis and effectiveness of ADT [21].

Now we focus on AHT. This study aims to report the prognosis of AR-V7 positive patients receiving AHT. AHT after RP may be considered as an effective treatment for patients with high-risk localized and locally advanced prostate cancer in China although it is recommended only for pN+ by current European Association of Urology (EAU) Guidelines [39, 40]. Moreover, in China, quite a few patients choose to receive AHT instead of adjuvant radiotherapy because of the fear of complications of radiotherapy.

The AR-V7 positive rates of nmHSPC vary substantially among the different studies [17, 21, 26–29] (range 1.6% from 91.8%), which may because of the differences in the TN stage, ethnicity and antibody. A study was reported by De Bono, Plymate, and colleagues [17], the authors did an excellent and professional work characterizing the AR-V7 antibody ab198394 (EPR15656 or EP343). In addition, to ensure that the protein detected by ab198394, Heng L et al. [25] carried out protein and RNA isolation followed by western blot and qPCR analysis on fresh tissue from selected prostate cancer patients. They found that AR-V7 was detected by ab198394 at the correct size and appeared as the major protein on western-blot in prostate cancer tissues, a finding consistent with the results in De Bono’s study when similar samples were assayed. Moreover, Kaczorowski A et al. [26] directly compared the two antibodies used for the immunodetection of AR-V7 (clones AG10008 and RM7) in a predominantly high-risk prostate cancer patient cohort. Although the overall rate of AR-V7 positive TMA cores was comparable (AG10008, 24.9%; RM7, 21%), the percentage agreement of identical staining intensities of positive cores was only 7%. Clearly, improvements in the detection of functional AR-V7 in prostate cancer are urgently needed. Our previous study reported that the AR-V7 positive rates of nmHSPC was 11.1% [21], which is lower than current study (19.1%). The difference may because of inclusion of lower T stage (<T2) and usage of biopsy tissue in previous study [21, 25]. Our results showed that AR-V7 was an independent risk factor for PFS, OS and CSS in high-risk prostate cancer patients who received adjuvant treatment. In subgroup analysis, our results showed AR-V7 status was an independent risk factor for OS and CSS in locally advanced disease, but not the localized disease. Xin et al. demonstrated that the presence of AR-V7-positive tumor cells is associated with an unfavorable prognosis for BCR-free survival in patients received adjuvant therapy [41]. The criteria for inclusion of these two studies are different. Only patients received adjuvant androgen-deprivation therapy after radical prostatectomy were selected for our study. Xin et al. collected all patients received adjuvant therapy which including neoadjuvant anti-hormonal therapy, adjuvant anti-hormonal or radiotherapy. Moreover, our study has more high-risk patients.

Despite recent medical advances in advanced prostate cancer, the development of systemic adjuvant therapy has remained relatively stagnant over the last few decades for patients with high-risk disease, consisting of only ADT. Novel hormonal therapies may provide oncologists with more efficacious drugs in the adjuvant setting, potentially leading to effective adjuvant therapy options for clinicians treating men with high-risk localized prostate cancer. Some retrospective cohort study demonstrated that adjuvant radiotherapy plus ADT was associated with improved OS compared to ADT alone (HR = 1.5) [42]. For AR-V7-positive prostate cancer patients, a novel therapeutic strategy is needed to improve treatment outcomes. Antonarakis and colleagues demonstrated that taxanes appear to be more efficacious than enzalutamide or abiraterone therapy in AR-V7-positive patients [43]. Furthermore, AR-V7-positive patients may also benefit from drugs which directly target AR-V7, such as ASC-J9, cisplatin, niclosamide, etc. [44].

### Limitations

First, because of the retrospective and observational nature and the limited cases of this study, selection bias may have occurred. Second, because all patients in the study were from one center in central China, caution should be taken in the generalization of our results to other populations. Third, no patients received RT followed by AHT were included in this study. Last but not least, some patients were pN0, who were not recommended to receive AHT by current EAU guideline. Prospective multicenter studies are urgently needed. Nevertheless, our findings encourage prospective studies to test the role of nuclear AR-V7 protein as a marker for aggressive tumor characteristics among high-risk patients.

## Conclusion

The presence of AR-V7 in prostate cancer tissue is independently associated with an unfavorable prognosis for PFS, OS and CSS in patients who received AHT. The expression of nuclear AR-V7 protein hence identifies a subset of tumors with remarkably aggressive growth characteristics among high-risk patients at the time of radical prostatectomy.

## Supplementary Information

**Additional file 1: Supplementary Table 1.** The AR-V7 positive rates of nmHSPC reported in published studies*. **Supplementary Table 2.** Univariate Cox analyses of PFS, OS and CSS in patients received AHT. Univariate Cox analyses of PFS, OS and CSS in patients received AHT.

## Data Availability

The data used to support the findings of this study are available from the corresponding author upon request.

## Abbreviations

AA: Anti-androgen
ADT: Androgen deprivation therapy
AHT: Adjuvant hormonal therapy
ARSi: Androgen receptor signal pathway inhibitor
AR-V7: Androgen Receptor Splice Variant 7
AR-FL: Androgen receptor full length
BCR: Biochemistry recurrence
CAB: Combined androgen blockade
CRPC: Castration-resistant prostate cancer
CSS: Cancer-special survival
CT: Computed tomography
ePLND: extended pelvic lymph node dissection
HR: Hazard ratio
HSPC: Hormone-sensitive prostate cancer
IHC: Immunohistochemical
LHRHa: Luteinizing hormone releasing hormone analogs
nmHSPC: nonmetastatic hormone-sensitive prostate cancer
OS: Overall survival
PFS: Progression-free survival
PSA: Prostate-specific antigen
PSAD: Prostate-specific antigen density
RCT: Randomized clinical trial
RP: Radical prostatectomy
RT: Radiotherapy
