# Supplementary material for: Androgen receptor splice variant 7 detected by immunohistochemical is an independent poor prognostic marker in men receiving adjuvant androgen-deprivation therapy after radical prostatectomy
Source: Biomark Res. 2021 Mar 31;9:23. doi: 10.1186/s40364-021-00276-x (PMC8011087; doi:10.1186/s40364-021-00276-x)
Supplement: Supplementary file 1 — Additional file 1: Supplementary Table 1. The AR-V7 positive rates of nmHSPC reported in published studies*. Supplementary Table 2. Univariate Cox analyses of PFS, OS and CSS in patients received AHT. Univariate Cox analyses of PFS, OS and CSS in patients received AHT. [file 40364_2021_276_MOESM1_ESM.docx]

**Supplementary Table 1. The AR-V7 positive rates of nmHSPC reported in published studies***

| Study | Journal | AR-V7 positive rate (total) | Antibody |
| --- | --- | --- | --- |
| Kallio HML, 2018 [28] | Br J Cancer | 91.8% (146) | RM7 |
| Qu Y, 2015 [29] | Sci Rep | 12% (100) | AG 10008 |
| Sharp A, 2019 [27] | J Clin Investig | 1.6% (3937) | RM7 |
| Li H, 2018 [21] | Mod Pathol | 11.1% (36) | ab198394 |
| Kaczorowski A, 2020 [26] | Cancer Treat Res Commun | 24.9% (514) | AG 10008 |
|  |  | 21% (520) | RM7 |
| Li H, 2021 [25] | Eur Urol | 21% (310) | ab198394 |

*****The expression of AR-V7 was detected by immunohistochemical.

nmHSPC：nonmetastatic hormone-sensitive prostate cancer

**Supplementary Table 2. Univariate Cox analyses of** **PFS, OS and CSS in patients received AHT.**

| Variables | Outcomes | Univariate analysis | |
| --- | --- | --- | --- |
|  |  | HR (95% CI) | P-value |
| Age at diagnosis (ref: <65.0 years)  Age≥65.0years | PFS | 1.06(0.50-2.27) | 0.876 |
|  | OS | 2.12(0.55-8.20) | 0.275 |
|  | CSS | 4.61(0.54-40.00) | 0.163 |
| Gleason score at diagnosis (ref: 6≤ G ≤7)  Gleason score ≥8 | PFS | 1.09(0.50-2.38) | 0.828 |
|  | OS | 1.77(0.51-6.13) | 0.367 |
|  | CSS | 3.48(0.64-18.87) | 0.150 |
| Total prostate-specific antigen (ng/ml) (ref: <20)  PSA≥20 | PFS | 0.57(0.26-1.21) | 0.143 |
|  | OS | 1.30(0.33-5.05) | 0.705 |
|  | CSS | 1.355(0.150-12.195) | 0.786 |
| Pathological T stage at diagnosis (ref: T_2_)  T3,4 | PFS | 2.87(1.31-6.29) | **0.008** |
|  | OS | 0.95(0.27-3.37) | 0.934 |
|  | CSS | 1.40(0.28-6.94) | 0.682 |
| Pathological N stage at diagnosis (ref: N0)  N1 | PFS | 1.45(0.66-3.15) | 0.357 |
|  | OS | 1.39(0.39-4.95) | 0.607 |
|  | CSS | 0.41(0.05-3.52) | 0.417 |
| AR-V7-positive (ref: AR-V7-negative) | PFS | 2.92(1.33-6.40) | **0.007** |
|  | OS | 4.03(1.17-13.89) | **0.028** |
|  | CSS | 7.81(1.42-43.48) | **0.018** |
| R1(ref: R0) | PFS | 2.40(1.05-5.49) | **0.038** |
|  | OS | 2.02(0.52-7.87) | 0.309 |
|  | CSS | 0.86(0.10-7.41) | 0.892 |

AHT= adjuvant hormonal therapy; PSA= prostate specific antigen; PFS= progression-free survival; OS= overall survival; CSS= cancer-special survival. P-values less than 0.05 are highlighted in bold.
